# Extraction-Free, Direct Determination of Caffeine in Microliter Volumes of Beverages by Thermal Desorption-Gas Chromatography Mass Spectrometry

**DOI:** 10.1155/2020/5405184

**Published:** 2020-04-01

**Authors:** Xianglu Peng, Melanie Brown, Paul Bowdler, Kevin C. Honeychurch

**Affiliations:** Department of Applied Sciences, University of the West of England, Frenchay Campus, Coldharbour Lane, Bristol BS16 1QY, UK

## Abstract

An extraction-free method requiring microliter (*μ*L) volumes has been developed for the determination of caffeine in beverages. Using a pyrolysis-gas chromatography mass spectrometry system, the conditions required for the direct thermal desorption-gas chromatography mass spectrometry (TD-GC/MS) determination of caffeine were optimised. A 5 *μ*L aliquot was introduced to the thermal desorption unit, dried, and thermally desorbed to the GC/MS. The response was linear over the range 10 to 500 *μ*g/mL (*R*^2^ = 0.996). The theoretical limit of detection (3 *σ*) was 0.456 *μ*g/mL. No interferences were recorded from endogenous beverage components or from commonly occurring drugs, such as nicotine, ibuprofen, and paracetamol. Replicate caffeine determinations on fortified latte style white coffee and Pepsi Max® gave mean recoveries of 93.4% (%CV = 4.1%) and 95.0% (%CV = 0.98%), respectively. Good agreement was also obtained with the stated values of caffeine for an energy drink and for Coca-Cola®. These data suggest that the method holds promise for the determination of caffeine in such samples.

## 1. Introduction

Caffeine is one of the most commonly consumed drugs, with a long history of usage [1], being commonly used to improve mental alertness and alleviate fatigue. It has been found to give a number of both beneficial and adverse effects on a wide range of conditions [2]. It is naturally found in a number of commonly consumed beverages such as in tea, coffee, and chocolate and is also commonly added to a wide range of both alcoholic and nonalcoholic drinks, medicines [3], and cosmetics [4] and is present in some electronic cigarette e-liquids [5]. It is reported that the majority of people regularly consumes caffeine [6, 7], and coffee has been reported as the second most commonly consumed drink after water [8]. The prevalence of caffeine in the society has also recently been highlighted by its occurrence in the majority of human blood donations [9].

Caffeine is one of the few drugs allowed in sport; its beneficial effects in disciplines that require endurance, such as cycling [10], triathlon [11], and soccer [12], have been reported. These benefits result not from the initial increase in heart rate and blood pressure but from the blocking of adenosine receptors responsible for the feelings of fatigue by similarly structured caffeine. Nevertheless, concerns have arisen regarding caffeine consumption by vulnerable groups, such as children [13, 14] or during pregnancy [15, 16]. Excessive caffeine consumption has been known to lead to the condition “caffeinism,” reported to be characterised by tinnitus, mood swings, diarrhoea, delirium, muscle tension, and tremors [17, 18]. In more extreme cases, a number of deaths have also been reported [19–21].

Caffeine levels in brewed beverages can exhibit wide variation depending on the source and the preparation method [22–25]. Even when purchased from the same vendor, they can vary by as much as 46% from day-to-day [23]. This can present an issue for those wishing to monitor their caffeine consumption for religious [26], sporting [10–12], or medical reasons [2, 15] and can be a problem for suppliers and manufactures [27].

Consequently, it is important to be able to determine caffeine levels in beverages, and a wide range of analytical methods have been utilised for its determination, including infrared [28] and ultraviolet spectroscopy [29]. Electrochemical approaches, such as amperometry and voltammetry [30], have also been reported. Recently, techniques such as laser desorption mass spectrometry [31] have also been reported; however, more commonly, high-performance liquid chromatography [9, 22–25, 32–34] or gas chromatography (GC) [35–41] is employed, and its application has recently been reviewed [42].

Although exhibiting a high melting point of 236.2°C [43], caffeine undergoes sublimation at the relatively low temperature of 178°C [43] making it amenable to quantification by GC without the need for derivatization. However, samples such as beverages offer a number of problems for analysis by GC such as the high aqueous content and the presence of proteins, sugars, and fats that make direct introduction of the sample difficult, and an extraction step is required. However, such extraction steps can be time consuming, labour-intensive, and lead to possible health and safety and disposal issues. The formation of emulsions in liquid extraction may also require further sample processing. Alternatively, solid phase extraction [39] can be less time consuming and is open to automation. However, this requires column conditioning and elution with organic solvents and some method of drawing solutions through the solid phase extraction cartridge.

A possible alternative to these approaches is the technique of thermal desorption-gas chromatography (TD-GC). This offers a number of possible advantages, both in terms of simplicity and speed and required sample size. Previously, a number of studies have investigated the possibility of utilising TD-GC for the determination of caffeine in both solution and for solids. However, these have either used a prior extraction step [35, 39–41, 44, 45] or have been qualitative studies [46]. In a recent study [47], we have shown that direct introduction of aqueous samples can be made without the need for tedious sample extraction procedures using a pyrolysis-gas chromatographic system. The technique also offers advantages in terms of health and safety, avoiding the need for toxic solvents and their disposal. To our knowledge, there have been no previous reports describing such an approach for the direct determination of caffeine in beverages. The aim of the present study was to investigate the development of a thermal desorption-gas chromatography mass spectrometry (TD-GC/MS) method for the extraction-free determination of caffeine in *μ*L volumes of beverages.

## 2. Materials and Methods

### 2.1. Chemicals and Reagents

All reagents were obtained from Sigma-Aldrich (Gillingham, UK) unless otherwise stated. Deionised water was obtained from a Purite RO200-Stillplus HP System, (Purite Oxon, UK) or a Sartorius Arium® mini Ultrapure Water System (Sartorius UK Ltd., Epsom, UK). Beverage samples were obtained from local commercial outlets. Separate stock solutions of caffeine (99.0%), lidocaine (≥99%), methanol (≥99.9%), ethanol (≥99.8%), acetonitrile (≥99.9%), nicotine (≥99%), paracetamol (99.0%), ibuprofen (≥99.8%), ethylene glycol (99.8%), 1,4-butandiol (99%), ɣ-butyrolactone (≥99.9%), 1,4-hydroquione (≥99.9%), phenol (≥99%), 1-naphthol (≥99%), and benzocaine (≥99%) were made by dissolving the appropriate mass in deionised water to give 500 *μ*g/mL solutions. Working standards were then made by further dilution with deionised water.

### 2.2. Instrumentation

Analytical standards and sample extracts were introduced via a CDS 5200 pyrolysis thermal desorption instrument (CDS Analytical, Oxford, PA). The CDS 5200 pyrolysis thermal desorption instrument conditions were as follows: drying temperature 80°C for 20 s; desorption temperature 250°C for 2 minutes. Transfer line 250°C was connected to the injector port operating in the split mode (10 : 1) of Agilent 6890 gas chromatography coupled to Agilent 5973 Network Mass Selective Detector. Chromatographic separations were undertaken using a He mobile phase, at a constant flow rate of 1.0 mL/min with a HP-5MS capillary column (15 m × 0.25 mm ID, 0.25 *μ*m film thickness, and 5% diphenyl–95% dimethylsiloxane phase). The GC oven temperature was maintained at 100°C for 2 minutes, then programmed to 280°C at 40°C/minute, and finally held isothermally for 3.0 minutes at this temperature. The injector was held at 240°C, and the transfer lines were at 330°C. The source temperature was at 200°C. Chromatograms were recorded in the full scan mode (m/z 50 to m/z 500). The pyrolysis probe tube (CDS Analytical, Oxford, PA) was cleaned between each sample or standard by heating to a temperature of 600°C for 10 s. The pyrolysis thermal desorption instrument was controlled using DCI software version 2.1.56 (CDS Analytical, Oxford, PA). Data processing and control of the GC/MS were undertaken using Enhanced ChemStation version D.00.01.27 (Agilent Technologies, USA).

### 2.3. Sample Preparation

Suitable aliquots (10 *μ*L to 100 *μ*L) of beverage samples were pipetted to a 1.0 mL Eppendorf vial. A 100 *μ*L aliquot of the internal standard solution (lidocaine, 500 *μ*g/mL in water) was then added. The sample was then diluted with sufficient deionised water to give a 500 *μ*L total volume.

### 2.4. Sample Analysis

A suitable aliquot of this solution (5 *μ*L) was pipetted into a pyrolysis tube containing a plug of glass wool (Fisher Scientific, Loughborough, UK) and placed into the pyrolysis probe tube. The remainder of the procedure was then carried out under the automated control of the CDS 5200 pyrolysis thermal desorption instrument. The pyrolysis tube containing the sample was first dried by heating at 80°C for 20 s. The probe was then inserted into the thermal desorption instrument and heated to 250°C in an inert atmosphere (He) to thermally desorb the sample components from the dried sample residue. The resulting vapour was then focused onto an integrated Tenax® cartridge and thermally desorbed to the gas chromatograph and the oven temperature program initiated. The pyrolysis probe was then removed, and the remaining extracted sample residue contained in the pyrolysis tube was removed by heating at 600°C for 10 s.

## 3. Results and Discussion

### 3.1. Effect of Sample Volume

**Figure 1:**
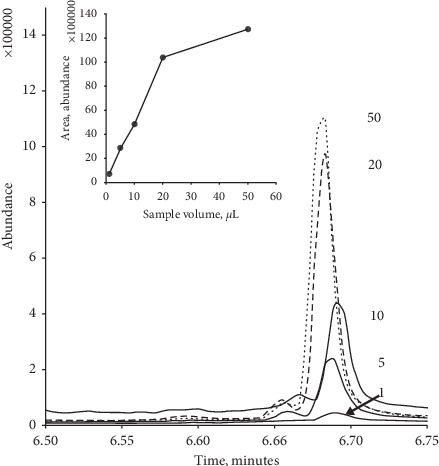
Effect of introduced sample volume on the resulting full-scan caffeine GC/MS response. Numbers represent sample volume in *μ*L. Insert plot of peak area vs. sample volume.

One major advantage of our approach is that large aqueous sample volumes can be readily introduced and then thermally dried before then being transferred to the chromatographic column. Consequently, we investigated the effect of sample volume over the range 1 *μ*L to 50 *μ*L on the resulting chromatographic peak for 23.5 *μ*g/mL caffeine solution. As can be seen from Figure 1, over the range 1 *μ*L to 20 *μ*L, a linear relationship with the resulting peak area (*R*^2^ = 0.997) for caffeine was obtained. However, at sample volumes above 20 *μ*L, the response began to plateau. Consequently, in further investigations, a sample volume of 5 *μ*L was utilised.

### 3.2. Choice of the Internal Standard

**Figure 2:**
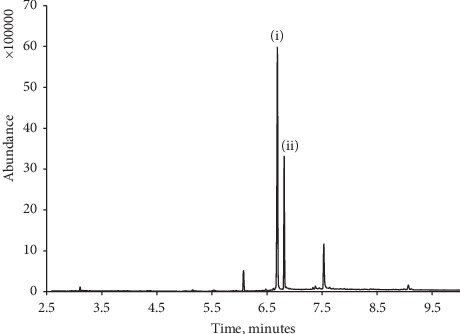
Typical chromatogram obtained for a 5 *μ*L sample of 117 *μ*g/mL caffeine (i) with lidocaine, internal standard (ii).

We investigated the possibility of utilising several compounds as an internal standard. Selection of these was based on the requirement that they are water soluble and hence soluble in the sample and should be thermally stable, allowing for their determination by TD-GC/MS. Consequently, we investigated 1,4-hydroquinone as previously proposed by Lin et al. (2000), *γ*-butyrolactone, phenol, 1-naphthol, lidocaine, and benzocaine. Our investigations found that, upon storage in aqueous solution, 1,4-hydroquinone formed purple colour, presumably due to the formation of the corresponding quinhydrone [48] leading to a question regarding its suitability. We next investigated *γ*-butyrolactone as an internal standard, but further investigations showed this to be naturally present in a number of beverages [49]. Unlike phenol, lidocaine, 1-naphthol, and benzocaine were all found to exhibit good chromatographic behaviour. However, both 1-naphthol and benzocaine eluted at retention times notably shorter than caffeine. As shown in Figure 2, lidocaine was found to elute at a retention time close to caffeine and hence concluded to be the best choice of the internal standard as it should have very similar chromatography properties. Consequently, we employed this as an internal standard in further investigations.

### 3.3. Effect of Chromatographic Starting Temperature

A sufficient low enough initial chromatographic starting temperature is required to focus target analytes on the analytical column. However, the use of too low initial starting temperature can lead to excessive run times, and in addition, the time required for cooling back down to the initial starting temperature between runs can become excessive. Consequently, it is important to identify the optimum chromatographic starting temperature. The parameter was studied over the temperature range 60°C to 180°C. At temperatures between 60°C and 100°C, no decrease in the resulting peak height for caffeine was observed. However, at chromatographic starting temperatures between 120°C and 180°C, the resulting peak height was found to decrease threefold. Consequently, in further investigations, an initial starting temperature of 100°C was used. We utilised a fast temperature ramp of 40°C/min so as to gain a short runtime. Similarly, a relatively short isothermal period of 3 minutes at 280°C was also chosen to facilitate this as well. We found this possible as no carryover issues were observed.

### 3.4. Precision, Calibration Plot, Limit of Detection, and Quantification

Standard solutions containing caffeine in the concentration range 10–500 *μ*g/mL were prepared in deionised water and determined by the optimized TD-GC/MS procedure. Quantification was based on peak area measurements taken from the total ion chromatogram. Using lidocaine as an internal standard, the calibration plot was found to be linear over the range studied, with an *R*^2^ value of 0.996. A coefficient of variation of 3.1% was obtained for replicate runs of a 23.5 *μ*g/mL caffeine standard (*n* = 4). The theoretical limit of detection, based on 3 *σ*, was calculated as 0.459 *μ*g/mL of caffeine, and the limit of quantification based on 10 *σ* was 1.53 *μ*g/mL for a 5 *μ*L sample. This is an improvement on the detection limits reported previously [50, 51].

### 3.5. Studies of Possible Interferences

Ethylene glycol, 1,4-butandiol, 1,2-butandiol, and gamma-butyrolactone were all found to elute with retention times (RTs) shorter than 5 minutes and were hence well resolved from both caffeine (RT = 6.69 minutes) and the internal standard (RT = 6.81 minutes). Similarly, nicotine (RT 5.1 minutes) at 130 *μ*g/mL and ibuprofen (RT 6.0 minutes) at 127 *μ*g/mL were also found which did not interfere. Paracetamol, methanol, ethanol, and acetonitrile were not detectable and did not interfere with the determination of caffeine or the internal standard.

## 4. Analytical Application

**Figure 3:**
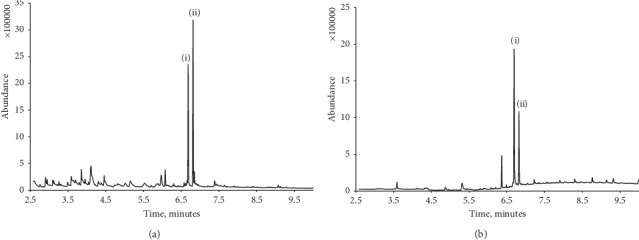
Typical chromatogram obtained for the latte style white coffee sample (a) and Pepsi Max® (b). Caffeine (i) with lidocaine, internal standard (ii).

To assess the performance of the optimised TD-GC/MS method, replicate determinations of caffeine in two fortified and unfortified latte style white coffee and Pepsi Max® were undertaken. Aliquots of these were pipetted to separate Eppendorf tubes, and the appropriate volume of the internal standard was then added and the solution was diluted with deionised water. A 5 *μ*L aliquot of this was then introduced to TD-GC/MS and examined using the optimised conditions.  Figure 3 shows representative chromatograms for the two unfortified beverage samples. The extracts showed well-defined signals for caffeine and the internal standard, lidocaine. The mean caffeine concentration calculated for the Pepsi Max® sample was found to be 130 *μ*g/mL (%CV = 3.0%), in good agreement with the quoted caffeine concentration of 129 *μ*g/mL [52].

Aliquots of the latte style white coffee and the Pepsi Max® sample were then fortified to have an overall caffeine concentration of 440 *μ*g/mL and 317 *μ*g/mL caffeine by the addition of 187 *μ*g/mL and 200 *μ*g/mL, respectively. Mean percentage recoveries of 93.4% (%CV = 4.1%) and 95% (%CV = 0.98%) based on five replicate samples were obtained. These data demonstrate that the proposed method has promise for the determination of caffeine in beverage samples. Mean percentage recoveries of 93.4% (%CV = 4.1%) and 95.0% (%CV = 0.98%) were obtained for latte style coffee and Pepsi Max®, respectively.

**Figure 4:**
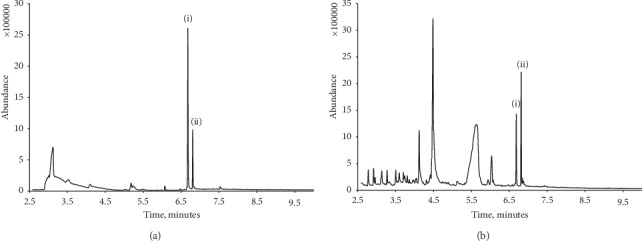
Typical chromatogram obtained for the energy drink (a) and Coca-Cola® (b). Caffeine (i) with lidocaine, internal standard (ii).

Figure 4(a) shows a typical chromatogram obtained for an energy drink (Monster Energy Absolutely Zero, Monster Beverage Corporation). Investigations using the optimised conditions gave a caffeine concentration of 301 *μ*g/mL, a value in close agreement with the stated label value of 300 *μ*g/mL. Two well-defined peaks for caffeine (RT = 6.69 minutes) and the internal standard, lidocaine, (RT = 6.81 minutes) were obtained. A broader peak with a retention time of 3.20 minutes was also recorded from its mass spectra concluded to result in the presence of glycerol in the beverage sample. Further investigations of the caffeine concentration of Coca-Cola® (Figure 4(b)) were undertaken, and a mean concentration of 96.9 *μ*g/mL was obtained. This was in close agreement with the literature caffeine concentration quoted for this beverage, 97 *μ*g/mL given by the manufacturer [53]. The chromatogram showed the presence of a number of peaks resulting from sucrose which were found not to interfere with the determination of caffeine or the internal standard.

## 5. Conclusions

Our extraction-free method for the determination of caffeine in beverages requires very little sample preparation, needing only dilution with water and addition of the internal standard. This offers a number of advantages over commonly reported methods for the determination of caffeine which require some form of sample clean-up or extraction step. We believe that the approach developed here could form the basis of a generic approach for the analysis of other drugs. In future studies, we plan to investigate this further. As far as we are aware, our report is the first to describe the use of a TD-GC/MS assay for the detection of caffeine.

With further investigation, it should be possible to develop the method further for the direct determination of caffeine in solid samples. We will also study the possibility of applying the developed method for the determination of caffeine in blood and other biological samples.

The assay was free from interferences from common beverage components or other common drugs. Use of full-scan mass spectrometry allows for better peak identification and the identification of unknown sample components. It should be noted that both improved selectivity and sensitivity would be readily obtainable via the application of selective ion monitoring or even by simple ion subtraction in postrun data processing. The small volumes of beverage (*μ*L) utilised offer advantages for health and safety of the analyst and in biomedical, food, and beverage investigations and to forensic science, where obtaining large samples volumes can be a problem.

## Data Availability

All related data are present in the submitted publication.
